# Comparative effectiveness of lazertinib in patients with EGFR T790M-positive non-small-cell lung cancer using a real-world external control

**DOI:** 10.1038/s41598-024-65220-z

**Published:** 2024-06-25

**Authors:** Ha-Lim Jeon, Meesong Kwak, Sohee Kim, Hye-Yeon Yu, Ju-Young Shin, Hyun Ae Jung

**Affiliations:** 1 School of Pharmacy and Institute of New Drug Development, Jeonbuk National University, Jeonju, Jeonbuk Republic of Korea; 2 School of Pharmacy, Sungkyunkwan University, 2066, Seobu-ro, Jangan-gu, Suwon-si, Gyeonggi-do Republic of Korea; 3 Yuhan Corporation, Seoul, Republic of Korea; 4 Division of Hematology-Oncology, Department of Medicine, Samsung Medical Center, Sungkyunkwan University School of Medicine, 81 Irwon-ro, Gangnam-gu, Seoul, 06351 Republic of Korea; 5 Department of Biohealth Regulatory Science, Sungkyunkwan University, Suwon, Republic of Korea; 6 Samsung Advanced Institute for Health Sciences and Technology (SAIHST), Sungkyunkwan University, Seoul, Republic of Korea

**Keywords:** Lazertinib, Osimertinib, External control, Comparative effectiveness, *EGFR* T790M-positive non-small-cell lung cancer, Non-small-cell lung cancer, Targeted therapies

## Abstract

Lazertinib is a recently developed third-generation epidermal growth factor receptor (EGFR)-tyrosine kinase inhibitors used for patients with advanced *EGFR* T790M-positive non-small-cell lung cancer. We evaluated the effectiveness of lazertinib compared with osimertinib using an external control. We obtained individual patient data for the lazertinib arm from the LASER201 trial and the osimertinib arm from registry data at the Samsung Medical Center. In total, 75 and 110 patients were included in the lazertinib and osimertinib groups, respectively. After propensity score matching, each group had 60 patients and all baseline characteristics were balanced. The median follow-up duration was 22.0 and 29.6 months in the lazertinib and osimertinib group, respectively. The objective response rate (ORR) were 76.7% and 86.7% for lazertinib and osimertinib, respectively (*p* = 0.08). The median progression-free survival (PFS) was 12.3 months (95% confidence interval [CI] 9.5–19.1) and 14.4 months (95% CI 11.8–18.1) for the lazertinib and osimertinib group, respectively (hazard ratio [HR] 0.97; 95% CI 0.64–1.45, *p* = 0.86). The median overall survival with lazertinib was not reached and that with osimertinib was 29.8 months (HR 0.44; 95% CI 0.25–0.77, *p* = 0.005). Our study suggests that lazertinib has an ORR and PFS comparable to those of osimertinib and has the potential for superior survival benefits.

## Introduction

Currently, two treatment options are available for patients with epidermal growth factor receptor (*EGFR*) T790M-positive non-small-cell lung cancer (NSCLC) who experienced disease progression following first- and second-generation EGFR-tyrosine kinase inhibitor (TKI) therapies in South Korea. Lazertinib (Leclaza^®^) is a recently developed third-generation EGFR-TKI^1,2^. LAZER201, a phase 1/2 single-arm study of lazertinib, showed that the objective response rate (ORR) was 55.3% and the median progression-free survival (PFS) was 11.1 months, which were evaluated by a blinded independent review (ORR of 72.4% and median PFS of 12.4 months by investigators)^3^. Another option is osimertinib (Tagrisso^®^), which is the first-approved third-generation EGFR-TKI and showed the ORR and median PFS of 70.1% and 10.1 months, respectively, in the AURA3 study^4^. Since both treatments have demonstrated excellent safety profiles, evidence on comparative effectiveness is needed to help oncologists decide the ideal treatment; however, there was no direct head-to-head study comparing the two drugs. Furthermore, the absence of data on the efficacy of lazertinib compared with that of the control group, including osimertinib or platinum-based chemotherapy, makes the comparison of the two treatments more challenging.

An external control arm is a good alternative in this situation. An external control is the comparator arm with no treatment or standard of care drawn from another population in different settings^5,6^. Since the background rates of outcomes cannot be adjusted in uncontrolled single-arm studies, a single-arm study with external control is considered to have a higher quality of evidence than those without^5,7^. Possible sources of external control are patient-level data from previous clinical trials and real-world data^8^. Therefore, in this study, we conducted an external control study to compare the effectiveness of lazertinib with that of osimertinib using individual patient data from the LASER201 study and the patient registry of a tertiary hospital in South Korea.

## Materials and methods

### Study population and data collection

Individual patient data (IPD) for the lazertinib arm were obtained from the LASER201 study (ClinicalTrials.gov Identifier: NCT03046992). LASER201 is a multicenter, open-label, phase 1/2, single-arm study that enrolled patients with locally advanced or metastatic *EGFR* T790M-positive NSCLC who had disease progression after prior therapy with an EGFR-TKI from November 27, 2017 to May 10, 2019^1^. Full details on the eligibility criteria and variables collected were described elsewhere^1^. Data cut-off for PFS and overall survival (OS) was January 8, 2021 and April 8, 2022, respectively.

As an external comparator, data from the osimertinib arm were extracted from real-world data of the Samsung Medical Center (SMC). Patients with locally advanced or metastatic *EGFR* T790M-positive NSCLC who had disease progression after prior therapy with an EGFR-TKI and started osimertinib between December 1, 2017, and May 31, 2019, were included. The SMC database includes sociodemographic information (e.g., age, sex, and smoking status) and various clinical information (e.g., Eastern Cooperative Oncology Group (ECOG) performance status, tumor histology, type of *EGFR* mutation, presence of brain metastasis, clinical stage at diagnosis, number of prior systemic therapies, type of prior EGFR-TKIs, number of previous EGFR-TKIs, results of laboratory tests, and history of other comorbidities). The data cutoffs for PFS and OS were the same as those for the lazertinib group (January 2021 for PFS and April 2022 for OS).

The same inclusion criteria in LASER201 were used for the osimertinib arm: (1) *EGFR* T790M-positive NSCLC and progression despite previous first- or second-generation EGFR-TKI therapies, (2) age ≥ 20 years, (3) ECOG 0–1, and (4) at least one measurable extracranial lesion. Among exclusion criteria of study subjects in LASER201, all applicable criteria in the SMC database were used. Patients who have the following history before the first use of osimertinib were excluded: (1) use of an investigational product within 30 days, (2) use of other EGFR-TKIs within 8 days, (3) cytotoxic chemotherapy or other anticancer drugs within 14 days, (4) major surgery within 4 weeks, (5) radiotherapy with a wide field within 4 weeks or a limited field within 1 week, (6) EGFR-TKIs targeting T790M mutation, (7) comorbidities within 8 weeks (spinal cord compression, brain metastases with steroid treatment, intracranial hemorrhage, central nervous system (CNS) complications with resection or shunt placement, leptomeningeal metastasis, interstitial lung disease, radiation pneumonitis with steroid treatment, symptomatic congestive heart failure, cardiac arrhythmia, or myocardial infarction/unstable angina within 6 months), and 8) clinical laboratory reports within 4 weeks (absolute neutrophil count < 1.5 × 10⁹ cells/L, platelet count < 100 × 10⁹ cells/L, hemoglobin < 90 g/L, alanine aminotransferase or aspartate aminotransferase > 2.5 × the upper limit of normal [ULN] if no demonstrable liver metastases or > 5.0 × ULN in presence of liver metastases, total bilirubin > 1.5 × ULN if no liver metastases or > 3.0 × ULN in the presence of documented Gilbert’s syndrome [unconjugated hyperbilirubinemia] or liver metastasis, or serum creatinine > 1.5 × ULN with creatinine clearance less than 50 mL/min). Additionally, we excluded patients with *EGFR* mutations other than exon 19 del and exon 21 Leu858Arg, because the number of patients was too small, making it difficult to include this variable in a matching model.

### Outcome measures

The endpoints of interest were ORR, PFS, and OS. In the IPD of the lazertinib arm, we used the best overall response, progression, and death assessed by investigators, not an independent central review, as we believe that these were more comparable with those from real-world data. Responses were assessed using CT or MRI according to the Response Evaluation Criteria in Solid Tumors (version 1.1). ORR was defined as the proportion of patients with a complete response or partial response. PFS was defined as the time from the first drug administration to disease progression or death due to any cause, whichever occurred first. OS was defined as the time from the first drug administration to death from any cause. The durations of PFS and OS were censored at the last date when they were examined as event-free in patients without observed events. We also examined the distribution of subsequent anticancer therapy after progression and calculated the time to next treatment (TTNT) to investigate whether a significant discordance existed in sequential treatment after drug resistance.

### Statistical analysis

To increase comparability between the two groups, we established a 1:1 propensity score-matched cohort. The propensity scores of each individual were estimated using a logistic regression model adjusted for variables that were considered clinically important or showed a significant difference between the groups before propensity score matching.

The ORR and median PFS and OS were calculated before and after PSM. Kaplan–Meier curves for PFS and OS were generated for the matched cohort and compared using a log-rank test. Hazard ratios (HRs) and their 95% confidence intervals (CIs) for progression and death with lazertinib versus osimertinib were estimated using Cox proportional hazard regression models. The risks of progression and death were examined according to age, sex, type of *EGFR* mutation, and the presence of brain metastasis, which can significantly affect the prognosis of patients with NSCLC. For sensitivity analysis, we applied the propensity score weighting approach [inverse probability of treatment weighting and standardized mortality ratio weighting instead of the matching method.

All analyses were performed using SAS (version 9.4, SAS Institute, Cary, NC, USA). A p-value of 5% or lower was considered statistically significant.

### Ethical statement

This study was approved with a waiver of the requirement for informed consent from participants by the Institutional Review Board of Samsung Medical Center (SMC 2021-11-130-002) because we used anonymously collected data. All methods were performed following the relevant guidelines and regulations.

## Results

### Baseline characteristics

**Figure 1:**
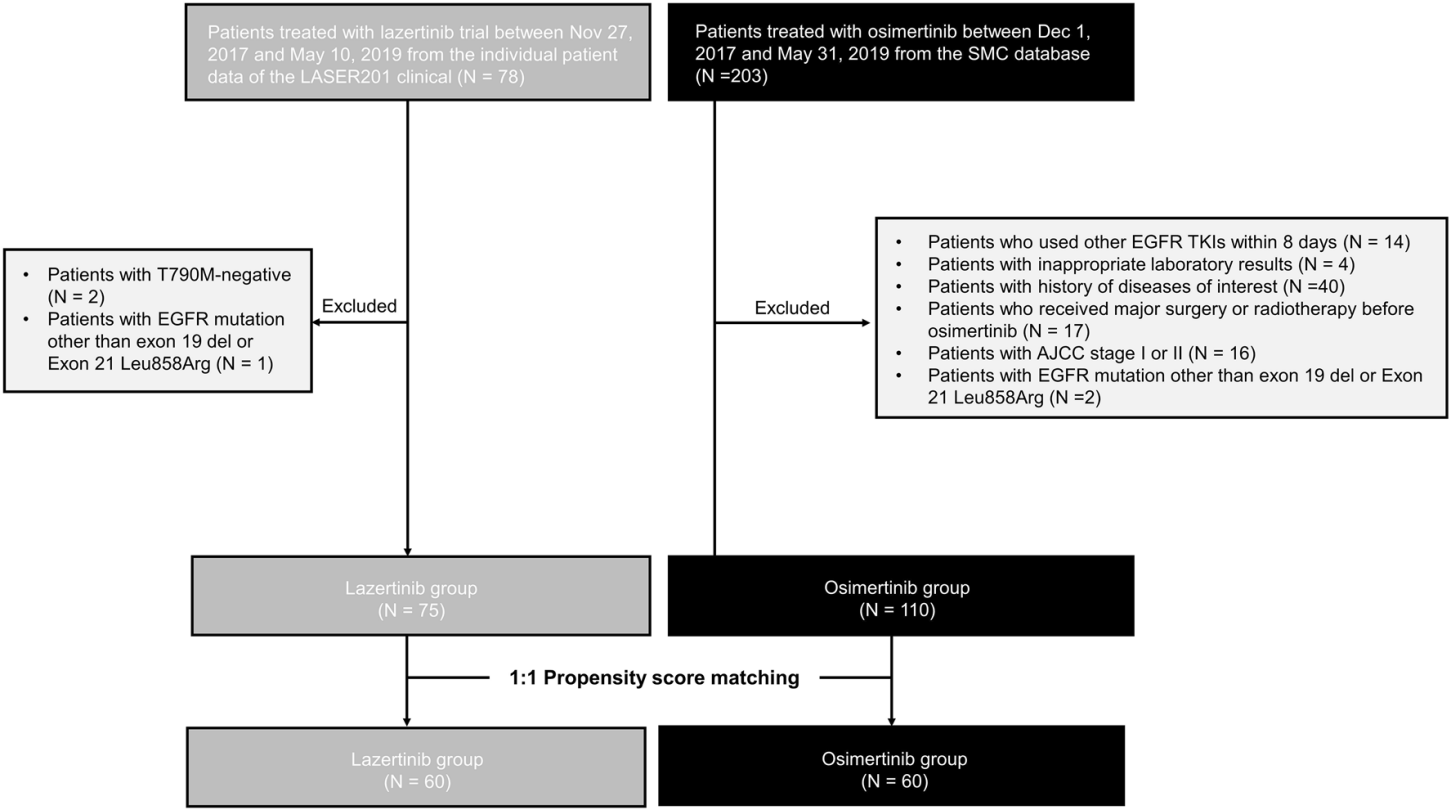
Flowchart for the identification and selection of the study cohort.

Between November 27, 2017, and May 10, 2019, 78 patients were enrolled and received lazertinib 240 mg. Two T790M-negative patients and one patient with *EGFR* mutations other than exon 19 del and exon 21 Leu858Arg were excluded; consequently, 75 patients remained (Fig. 1). In the SMC database, 203 patients started osimertinib treatment between December 1, 2017, and May 31, 2019. Among them, 93 patients were excluded as per protocol (14 with other EGFR-TKIs within 8 days, 4 with inappropriate laboratory results, 40 with a history of diseases of interest, 17 with major surgery or radiotherapy, 16 with American Joint Committee on Cancer stage I or II, and 2 with *EGFR* mutations other than exon 19 del or exon 21 Leu858Arg), and 110 patients were included in the final analysis.

The median follow-up duration for OS was 20.9 months [interquartile range (IQR) 8.8–35.8] in the lazertinib group and 29.3 months (IQR 17.9–36.8) in the osimertinib group. The median age of the lazertinib group was 62.0 years (range: 33–82), which was slightly but not significantly older than that of the osimertinib group (58.9 years, range: 29.5–88.1), and 52.0% and 40.0% of the patients in the lazertinib and osimertinib groups were male, respectively (Table 1). Regarding the type of *EGFR* mutation, 22 (29.3%) patients in the lazertinib group had an exon 21 Leu858Arg mutation, similar to that in the osimertinib group (30.9%). The proportion of patients with brain metastases was significantly higher in the lazertinib group (52.0%) than in the osimertinib group (27.3%) (*p* < 0.001). The mean number of prior EGFR-TKIs was 1.1 (standard deviation: 0.3) in both groups. The majority of patients received other EGFR-TKIs before lazertinib (92.0%) or osimertinib (83.7%). Additionally, 94.7% of patients in the lazertinib group and 94.6% in the osimertinib groups used prior EGFR-TKIs for over 6 months.

**Table 1:** Baseline characteristics of patients receiving lazertinib or osimertinib before and after propensity score matching.

Characteristic	Before-matching			After-matching^a^ (c-statistics = 0.680)		
	Lazertinib (N = 75)	Osimertinib (N = 110)	*p* value^b^	Lazertinib (N = 60)	Osimertinib (N = 60)	*p* value^b^
Age, years, median (range)	62.0 (33.0–82.0)	58.9 (29.5–88.1)	0.175	61.0 (33.0–82.0)	61.0 (38.3–80.5)	0.736
Sex, n (%)			0.107			1.00
Male	39 (52.0)	44 (40.0)		28 (46.7)	28 (46.7)	
Female	36 (48.0)	66 (60.0)		32 (53.3)	32 (53.3)	
Smoking, n (%)			0.202			0.590
Never smoking	40 (53.3)	69 (62.7)		35 (58.3)	32 (53.3)	
Ever smoking	35 (46.7)	41(37.3)		25 (41.7)	28 (46.7)	
Tumour histology, n (%)			0.045			0.159
Adenocarcinoma	71 (94.7)	110 (100.0)		58 (96.7)	60 (100.0)	
Squamous cell carcinoma	0 (0.0)	0 (0.0)		0 (0.0)	0 (0.0)	
Other	4 (5.3)	0 (0.0)		2 (3.3)	0 (0.0)	
Type of EGFR mutation, n (%)			0.819			0.847
Exon 21 Leu858Arg	22 (29.3)	34 (30.9)		16 (26.7)	15 (25.0)	
Exon 19 del	53 (70.7)	76 (69.1)		44 (73.3)	45 (75.0)	
Brain metastasis, n (%)			0.0006			0.317
Yes	39 (52.0)	30 (27.3)		24 (40.0)	21 (35.0)	
No	36 (48.0)	80 (72.7)		36 (60.0)	39 (65.0)	
AJCC stage, n (%)			0.130			0.706
IIIA/IIIB/IIIC	3 (4.0)	11 (10.0)		3 (5.0)	4 (6.7)	
IV	72 (96.0)	99 (90.0)		57 (95.0)	56 (93.3)	
Previous systemic therapy						
Platinum-based chemotherapy	23 (30.7)	29 (26.4)	0.523	20 (33.3)	19 (31.6)	0.819
Others	7 (9.3)	6 (5.5)	0.311	7 (11.7)	2 (3.3)	0.0002
Number of previous EGFR-TKIs, mean (SD)	1.1 (± 0.3)	1.1 (± 0.3)	0.513	1.1 (± 0.3)	1.1 (± 0.3)	0.754
Immediate prior EGFR-TKI, n (%)			0.339			0.847
Gefitinib or Erlotinib	51 (68.0)	75 (68.2)		41 (68.3)	42 (70.0)	
Afatinib	24 (32.0)	35 (31.8)		19 (31.7)	18 (30.0)	
Immediate prior treatment, n (%)						
EGFR-TKIs						
< 30 days	42 (56.0)	65 (59.1)	0.193	33 (55.0)	35 (58.3)	0.362
≥ 30 days	27 (36.0)	27 (24.6)		22 (36.7)	17 (28.3)	
Others	6 (8.0)	18 (16.4)		5 (8.3)	8 (13.3)	
Duration of prior EGFR-TKI, n (%)			0.971			0.067
< 6 months	4 (5.3)	6 (5.5)		3 (5.0)	5 (8.3)	
≥ 6 months	71 (94.7)	104 (94.6)		57 (95.0)	55 (91.7)	

^a^Age, sex, smoking history, presence of brain metastasis, type of EGFR mutation, prior treatment, and duration of prior EGFR-TKI treatment were used to calculate propensity scores.

^b^*p* values were calculated with t-test for continuous variables and chi-square test for categorical variable before matching and with paired t-test and McNemar test after matching. SD, standard deviation.

### Propensity score matching

PSM was conducted by using clinical variables including age (< 65 years and ≥ 65 years), sex, smoking history (never-smoker and ever-smoker), presence of brain metastasis, the type of EGFR mutation (exon 19 del and exon 21 Leu858Arg), prior EGFR-TKI (gefitinib or erlotinib and afatinib), and duration of prior EGFR-TKI (< 6 months and ≥ 6 months). Sixty patients were included in each group after the PSM. The median follow-up duration for OS was still approximately 8 months longer in the osimertinib group (29.6 months, IQR 16.7–36.5) compared with the lazertinib group (22.0 months, IQR 12.0–37.2). The median age was 61.0 years in both groups (range: 33.0–82.0 in the lazertinib group and 38.3–80.5 in the osimertinib group). Although significant differences were observed in tumor histology (*p* = 0.045) and brain metastasis (*p* < 0.001) before PSM, all covariates were well balanced after PSM (Table 1).

### Objective response rate and progression-free survival

**Figure 2:**
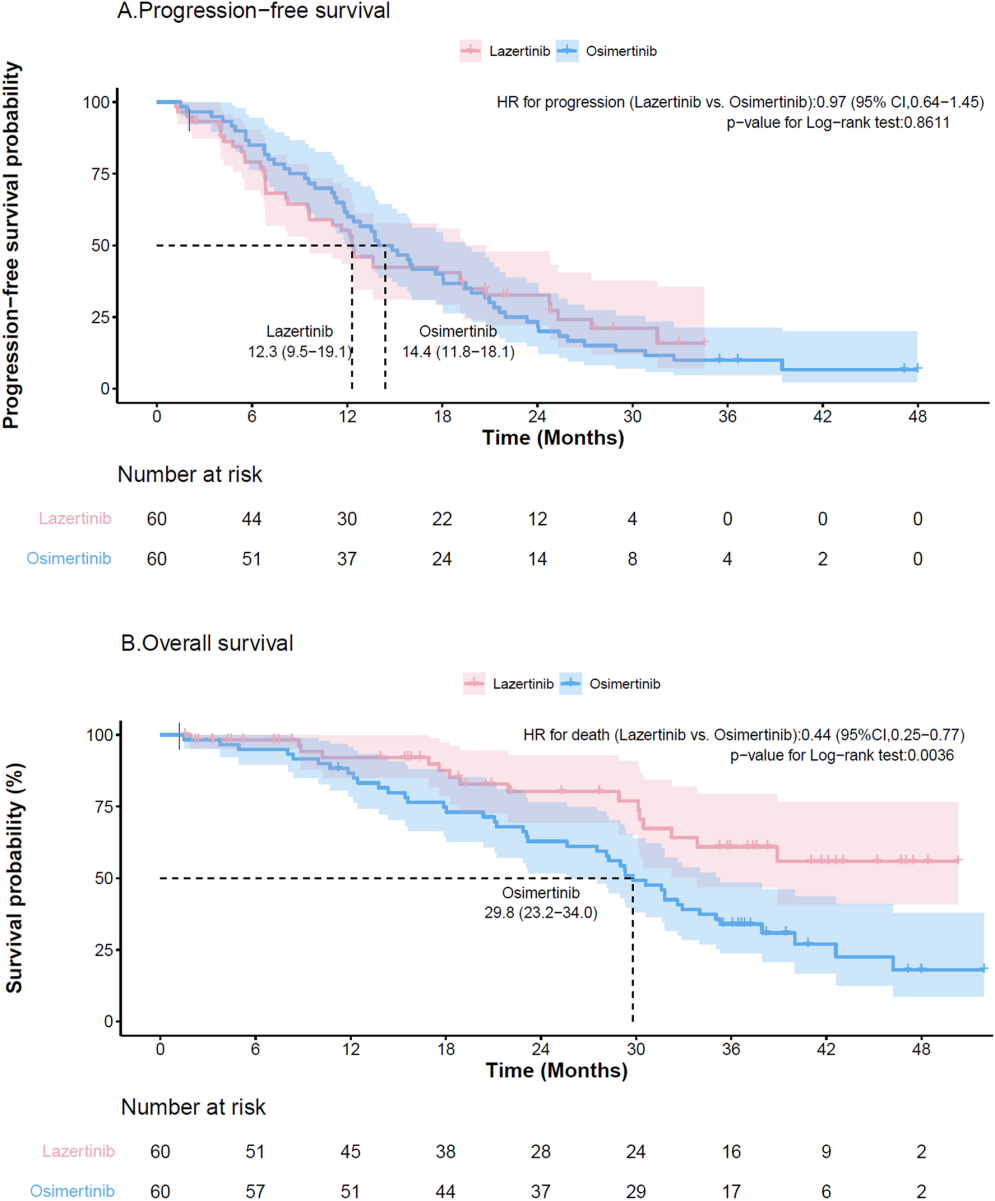
Kaplan–Meier curve for progression-free survival (**A**) and overall survival (**B**) of the lazertinib and osimertinib group after propensity score matching.

Before PSM, the ORR of osimertinib (84.5%) was higher than that of lazertinib (72.0%) (Table 2). The median PFS was 12.4 months (95% CI 9.6–17.7) in the lazertinib group and 15.7 months (95% CI 12.8–18.0) in the osimertinib group (*p* = 0.594). After PSM, ORRs in both groups increased slightly but remained higher for osimertinib (76.7% for lazertinib and 86.7% for osimertinib) (*p* = 0.078). Among the 60 patients, 42 (70%) and 55 (91.7%) patients progressed were identified in the lazertinib and osimertinib groups, respectively. The median PFS was still numerically longer in the osimertinib group (14.4 months; 95% CI 11.8–18.1) than in the lazertinib group (12.3 months; 95% CI 9.5–19.1; *p* = 0.861) (Fig. 2A). HR for progression in the lazertinib group was 0.97 (95% CI 0.64–1.45) compared with the osimertinib group.

**Table 2:** Objective response rate, overall survival, and progression free survival before and after propensity score matching.

Efficacy outcomes	Before-matching		After-matching^a^	
	Lazertinib (N = 75)	Osimertinib (N = 110)	Lazertinib (N = 60)	Osimertinib (N = 60)
ORR^b^, %	72.00	84.54	76.67	86.67
PFS				
Median, months (95% CI)	12.4 (9.6–17.7)	15.7 (12.8–18.0)	12.3 (9.5–19.1)	14.4 (11.8–18.1)
1-year PFS rate, %	56.3	61.8	56.5	61.7
2-year PFS rate, %	26.9	22.4	31.4	23.3
No. of events/Person-years	53/966.1	101/1905.8	42/824.0	55/1021.7
IR	0.055	0.053	0.051	0.054
Adjusted HR	1.10 (0.78–1.53)	1 (ref)	0.97 (0.64–1.45)	1 (ref)
OS				
Median, months (95% CI)	38.9 (30.2–NR)	29.6 (25.6–31.8)	NR	29.8 (23.2–34.0)
1-year OS rate, %	89.6	91.8	92.7	86.6
2-year OS rate, %	74.0	62.3	80.8	62.8
No. of events/Person-years	24/1678.3	79/3086.5	16/1452.0	43/1650.7
IR	0.014	0.026	0.011	0.026
Adjusted HR	0.58 (0.37–0.92)	1 (ref)	0.44 (0.25–0.77)	1 (ref)

*ORR* objective response rate, *OS* overall survival, *IR* incidence ratem, *HR* hazard ratio, *PFS* progression-free survival.

^a^Age, sex, smoking history, presence of brain metastasis, type of EGFR mutation, prior treatment, and duration of prior EGFR-TKI treatment were used to calculate propensity scores.

^b^In the lazertinib group, tumor responses judged by investigators, not independent central reviews, were used (i.e., unconfirmed ORR).

### Overall survival

Before PSM, the median OS was 38.9 months (95% CI 30.2-not reached) and 29.6 months (95% CI 25.6–31.8) in the lazertinib and osimertinib group, respectively (*p* = 0.019). The 1-year OS rates in the lazertinib and osimertinib arms were 89.6% and 91.8%, respectively (*p* = 0.62). After PSM, the number of death events was 16 (26.7%) and 43 (71.7%) in the lazertinib and osimertinib groups, respectively. The median OS with lazertinib was not reached, and that with osimertinib was 29.8 months (95% CI 23.2–34.0; *p* = 0.004). The 1-year OS rates with lazertinib was 92.7%, while that with osimertinib was 86.6% (*p* = 0.27). The Kaplan–Meier curves for OS in the lazertinib and osimertinib groups are shown in Fig. 2B. HR for death in the lazertinib group versus osmertinib group was 0.44 (95% CI 0.25–0.77) after PSM, which was statistically significant (*p* = 0.005). The median TTNT and the distribution of patients who received subsequent anticancer therapy in the matched groups were shown in supplementary Table S1. The median TTNT was 26.6 months (range, 13.8- not reached) with lazertinib and 15.7 months (range, 12.4–20.3) with osimertinib. Out of 60 patients, 24 (40%) and 39 (65%) received subsequent treatment in the lazertinib and osimertinib groups, respectively. The proportion of patients who received treatments with 3rd-generation EGFR TKI was higher in the lazertinib group than in the osimertinib group (15% vs. 8.4%).

### Subgroup and sensitivity analysis

**Figure 3:**
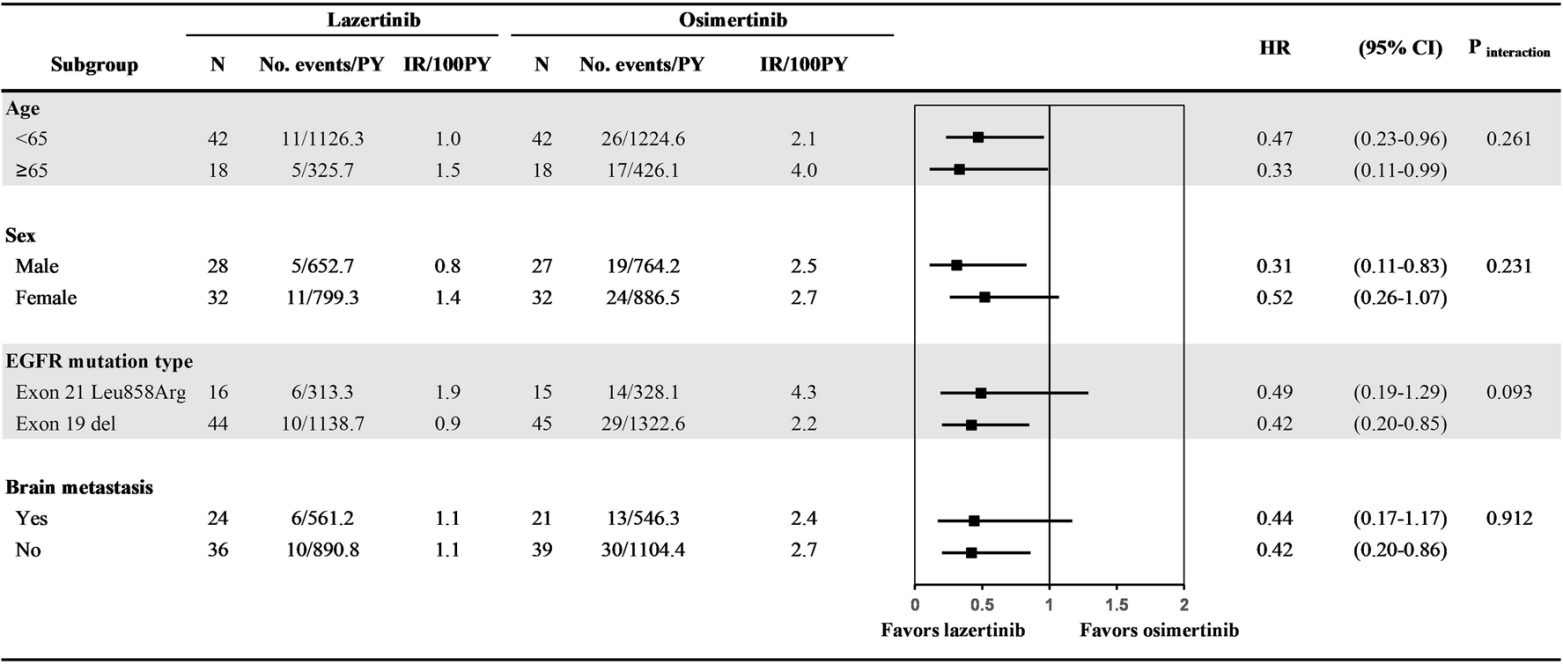
Subgroup analysis for overall survival in patients who received lazertinib or osimertinib after propensity score matching.

In the subgroup analyses, the overall trend in the risk of death was favorable for lazertinib (Fig. 3). Among them, the risk of death in lazertinib was significantly lower than that in osimertinib regardless of age (< 65 years: HR 0.47; 95% CI 0.23–0.96; *p* = 0.037; and ≥ 65 years: HR, 0.33; 95% CI 0.11–0.99; *p* = 0.049) and in subgroups male (HR 0.31; 95% CI 0.11–0.83; *p* = 0.019), with exon 19 del (HR, 0.42; 95% CI 0.20–0.85; *p* = 0.017), and without brain metastasis (HR, 0.42; 95% CI 0.20–0.86, *p* = 0.017). However, the risk of progression in all subgroups was not statistically significant (Supplementary Fig. S1). The results of sensitivity analysis using propensity score weighting methods were consistent with those of the main analysis (Supplementary Tables S2–4).

## Discussion

This study showed that lazertinib had ORR, PFS, and OS outcomes comparable to those of osimertinib before and after PSM. There was no significant difference in ORR and PFS between the two groups. The results of the subgroup analysis also revealed that the risk of death tended to be lower with lazertinib than with osimertinib, regardless of age group, sex, type of *EGFR* mutation, and brain metastasis status. We verified the robustness of these findings using sensitivity analyses.

The median PFS and OS in matched patients treated with osimertinib were estimated as 14.4 and 29.8 months, respectively. These results are similar to those of previous studies on osimertinib. In AURA3, a phase III trial investigating the efficacy and safety of osimertinib compared with platinum-pemetrexed among patients with *EGFR* T790M-positive locally advanced or metastatic NSCLC, the median PFS and OS were 10.1 and 26.8 months, respectively, in the osimertinib arm^4,9^. A study of real-world data of osimertinib from 19 medical centers reported the median PFS of 14.2 months and the median OS of 36.7 months^10^. Thus, it seems that the osimertinib group of this study demonstrated strong external validity.

Our study showed a 56% reduced risk of death with lazertinib compared to osimertinib, despite a similar risk of progression. Given the higher selectivity and potency of lazertinib compared to those of osimertinib in an in vitro study^11^, this finding may have resulted from the excellent efficacy of lazertinib. In particular, lazertinib showed superior intracranial activity compared to osimertinib in vivo and an intracranial ORR of 86% and a median intracranial PFS of 26.0 months in the LASER201 study^3,11^. The results of the subgroup analysis in the present study also support that lazertinib was likely to have a better survival benefit irrespective of brain metastasis. Although osimertinib showed good brain penetration and a good CNS objective response rate^12–14^, no significantly lower risk of death in patients with CNS metastasis in the osimertinib arm than in the platinum-pemetrexed arm was demonstrated in the subgroup analysis of the AURA 3 study (HR 1.19; 95% CI 0.79–1.83)^9^. Therefore, the potent CNS efficacy of lazertinib may contribute to the prolonged OS. Furthermore, the potentially better efficacy of lazertinib in the exon 21 Leu858Arg mutation could have led to a longer OS compared to osimertinib. In the subgroup analysis, lazertinib showed a remarkable point estimate of HR (0.49, 95% CI 0.19–1.29) in patients with exon 21 Leu858Arg mutation although CI was wide due to the small sample size. Previous studies reported that patients with exon 21 Leu858Arg have a worse prognosis than those with exon 19 del after anticancer therapies, including osimertinib^15–19^. Our results suggest that lazertinib may be more effective in patients with exon 21 Leu858Arg mutation and should be considered first for treatment.

However, a cautious interpretation of the longer OS is needed because of the considerable differences in the clinical settings of the groups. As shown in the Kaplan–Meier curves, many participants in the lazertinib trial dropped out within 2 years after treatment initiation due to withdrawal by the subject or loss to follow-up, whereas few patients did so in the registry data. In the same context, the median follow-up duration for OS was shorter in the lazertinib group than that in the osimertinib group. This could be attributed to the differential way death records were collected in the two databases; death could not be captured once a patient withdrew consent in the clinical trial, whereas death records in the SMC data was derived from the national cancer registry. This unequal early dropout rate and short follow-up duration would work in favor of lazertinib, especially in terms of long-term outcomes, as the investigators lost the chance to identify additional deaths in participants. Furthermore, differences in the distribution of subsequent treatments that patients underwent after progression between the two groups may have influenced the favorable results in OS for lazertinib. In the lazertinib group, 9 patients (15%) received osimertinib as a next-line treatment, while 4 patients (8.4%) received osimertinib plus savolitinib and only one patient (1.7%) received lazertinib plus amivantamab in the osimertinib group. However, 15 of 42 patients who experienced progression in the lazertinib group used lazertinib as a treatment beyond progressive disease, with the median duration of the treatment was 158 days (Supplementary Table S5). Considering the longer TTNT and the higher proportion of patients who maintained the treatment beyond progressive disease in the lazertinib group compared to the osimertinib group, the effect of subsequent treatment on the longer OS might not be considerable.

This study had several limitations. We attempted to make the osimertinib group as similar as possible to the lazertinib group by applying the same eligibility criteria; however, some criteria were unavailable in the retrospective real-world data. In addition, residual confounding factors may have originated from different clinical settings. Patients’ psychological status, measurement methods or techniques, standards of clinical judgement, and other prognostic factors can differ between the two settings. In our study, the different response assessment intervals (6 weeks for the lazertinib arm and 2–3 months for the osimertinib arm) may have affected the results. As mentioned above, different follow-up durations for OS due to different methods of collecting death information also impede a clear interpretation of OS differences. In addition, the small sample size and data from a single institute impede generalization of the results. Although we used all IPD of LASER201 and real-world data of SMC, which is a hospital with the largest data of NSCLC patients, further confirmative studies with a large population should be conducted. Lastly, we only evaluated the effectiveness of the drug, even though the safety of the drug is also an important factor when selecting a drug. Therefore, a study comparing the safety of the two drugs in a real-world setting needs to be conducted in the future.

Despite these limitations, this study has several important implications. We produced the first comparative evidence on the efficacy of lazertinib as a second-line or more EGFR-TKI at a time when there is no other study with direct, randomized head-to-head comparisons. Many researchers agree that studies with external comparators cannot substitute randomized studies with a direct comparison^20–22^. Nevertheless, our findings would provide helpful insights on the novel therapy to policymakers and practitioners since single-arm studies inherently have methodological limitations regarding interpreting the results^23^. We also present limitations of the study that should be considered and discussed for the appropriate regulatory use of this study design in the future. To generate valid comparative evidence using real-world data, regulatory agencies may need to develop guidelines for the uniform collection of real-world data, such as registries or electronic health records^24^.

In conclusion, our study strengthens the evidence on the comparable efficacy of lazertinib with osimertinib and the potential beneficial effect on the survival in patients with advanced *EGFR* T790M-positive NSCLC who experienced disease progression following EGFR-TKI, which was shown in a single-arm trial. However, the small sample size and different clinical settings between the groups hinder the generalizability of the results. A large-scale, controlled study is required to confirm these findings.

### Supplementary Information

Supplementary Information.

## Data Availability

The data analyzed in this study are available from Yuhan Corporation and Samsung Medical center. Restrictions apply to the availability of these data, which were used under license for this study. Data are available from the authors upon reasonable request with the permission of Yuhan Corporation and Samsung Medical center.
