# Supplementary material for: Comparative effectiveness of lazertinib in patients with EGFR T790M-positive non-small-cell lung cancer using a real-world external control
Source: Sci Rep. 2024 Jun 25;14:14659. doi: 10.1038/s41598-024-65220-z (PMC11199632; doi:10.1038/s41598-024-65220-z)
Supplement: Supplementary file 1 — Supplementary Information. [file 41598_2024_65220_MOESM1_ESM.docx]

**Comparative effectiveness of lazertinib in patients with EGFR T790M-positive non-small-cell lung cancer using a real-world external control**

Ha-Lim Jeon, Meesong Kwak, Sohee Kim, Hye-Yeon Yu, Ju-Young Shin_,_ Hyun Ae Jung

**Supplemental Online Content**

**Supplementary Figure S1.** Subgroup analysis of progression-free survival in patients who received lazertinib or osimertinib after propensity score matching.

**Supplementary Table S1.** Time to next treatment and the distribution of patients who received subsequent anticancer therapy after propensity score matching.

**Supplementary Table S2.** Overall survival and progression-free survival before and after inverse probability of treatment weighting and standardized mortality ratio weighting using propensity scores.

**Supplementary Table S3.** Subgroup analysis of the risk of death in patients who received lazertinib or osimertinib after inverse probability of treatment weighting using propensity scores.

Supplementary Table S4. Subgroup analysis of the risk of progression in patients who received lazertinib or osimertinib after inverse probability of treatment weighting using propensity scores.

**Supplementary Table S5**. The number of patients who maintained lazertinib treatment beyond progressive disease and duration of the treatment in the lazertinib group.


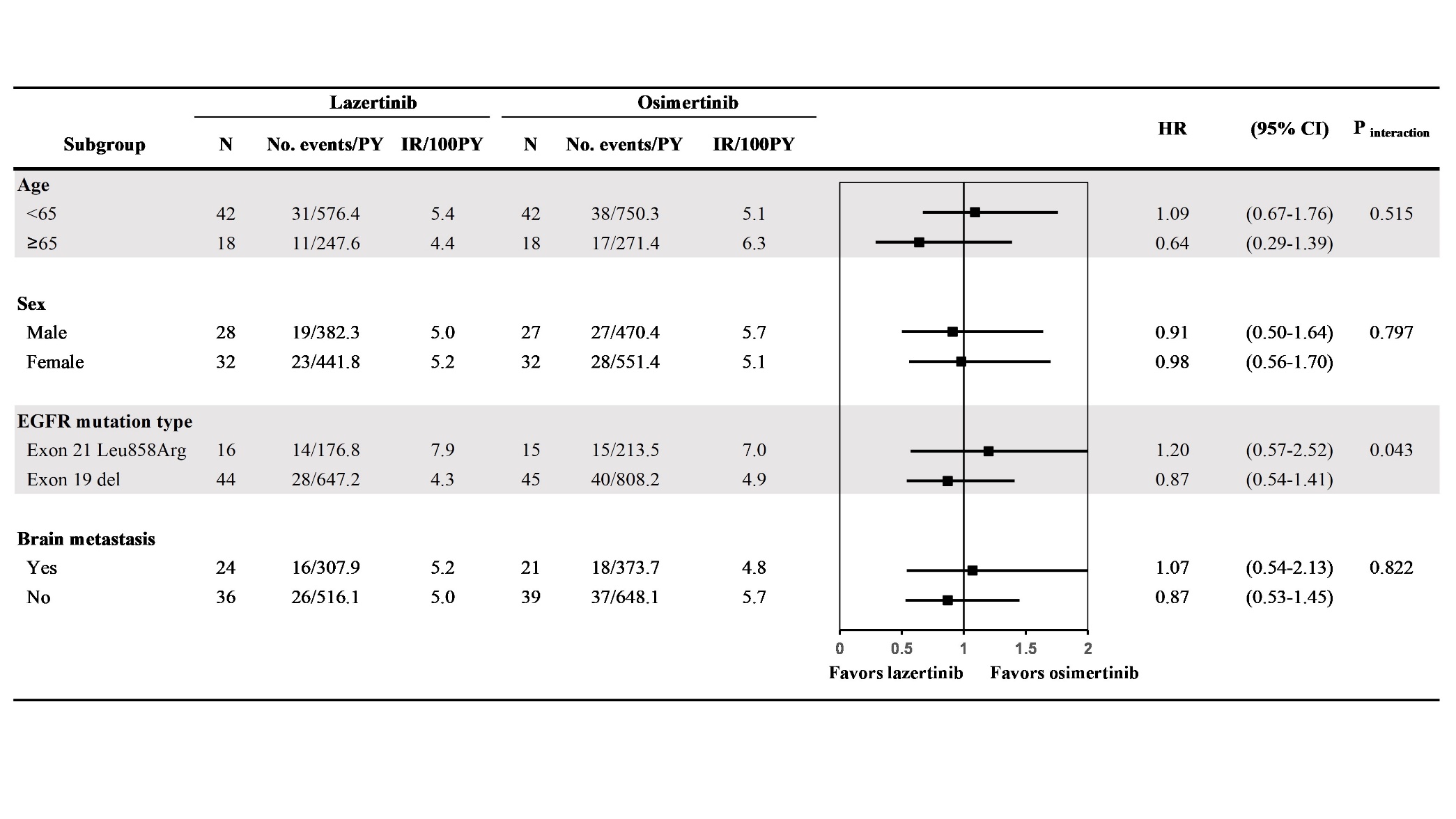
**Supplementary Figure S1.** Subgroup analysis of progression-free survival in patients who received lazertinib or osimertinib after propensity score matching.

**Supplementary Table S1.** Time to next treatment and the distribution of patients who received subsequent anticancer therapy after propensity score matching.

| **Efficacy Outcomes** | **Lazertinib (N = 60)** | **Osimertinib (N = 60)** |
| --- | --- | --- |
| ***TTNT***^a^ |  |  |
| Median, months (range) | 26.6 (13.8-NR) | 15.7 (12.4-20.3) |
| No. of events / Person-years | 28/1143.8 | 53/1064.2 |
| IR | 0.024 | 0.050 |
| Adjusted HR | 0.53 (0.34-0.82) | 1 (ref) |
|  |  |  |
| ***Subsequent anticancer therapy, n(%)*** | 24 (40) | 39 (65) |
| Platinum-based chemotherapy | 12 (20) | 23 (38.3) |
| Pemetrexed or gemcitabine | 2 (3.3) | 4 (6.7) |
| Osimertinib | 9 (15) | - |
| Osimertinib+Savolitinib | 0 (0) | 4 (6.7) |
| Lazertinib+Amivantamab | 0 (0) | 1 (1.7) |
| Immunotherapy (Pembrolizumab, Atezolizumab) | 0 (0) | 5 (8.3) |
| Investigational drugs | 1 (1.7) | 2 (3.3) |
|  |  |  |
| ***Treatment beyond progression^b^, n(%)*** | 15 (25) | 2 (3.3) |

^a^ TTNT was defined as the time between index date [first multiple dosing date] and the earliest date between starting a next line of treatment, death, migration or end of data availability [8 Apr 2022]. In the TTNT analysis, death was considered as competing event.

^b^ Lazertinib or osimertinib was administered more than 21 days after disease progression.

TTNT, Time to next treatment; IR, Incidence Rate; HR, Hazard ratio; PFS, Progression-free survival.

**Supplementary Table S2.** Overall survival and progression-free survival before and after inverse probability of treatment weighting and standardized mortality ratio weighting using propensity scores.

| **Efficacy Outcomes** | **Before-weighting** | | **After-weighting** | |
| --- | --- | --- | --- | --- |
|  | **Lazertinib (N = 75)** | **Osimertinib (N = 110)** | **Lazertinib** | **Osimertinib** |
| ***IPTW*** |  |  |  |  |
| HR for OS | 0.58 (0.37-0.92) | 1 (ref) | 0.53 (0.39-0.72) | 1 (ref) |
| HR for PFS | 1.10 (0.78-1.53) | 1 (ref) | 1.03 (0.82-1.29) | 1 (ref) |
| ***SMRW*** |  |  |  |  |
| HR for OS | 0.58 (0.37-0.92) | 1 (ref) | 0.58 (0.36-0.94) | 1 (ref) |
| HR for PFS | 1.10 (0.78-1.53) | 1 (ref) | 1.04 (0.72-1.49) | 1 (ref) |

IPTW, inverse probability of treatment weighting; HR, Hazard ratio; OS, Overall survival; PFS, Progression-free survival; SMRW, standardized mortality ratio weighting.

**Supplementary Table S3.** Subgroup analysis of the risk of death in patients who received lazertinib or osimertinib after inverse probability of treatment weighting using propensity scores.

| Variable | | Lazertinib | | | | Osimertinib | | | | Crude HR | p for interaction |
| --- | --- | --- | --- | --- | --- | --- | --- | --- | --- | --- | --- |
|  |  | N | No of event | Person-year | IR | N | No of event | Person-year | IR |  |  |
| Age group | < 65 | 48 | 13 | 1201.1 | 0.0108 | 79 | 51 | 2348.1 | 0.0217 | 0.54 (0.37-0.80) | 0.029 |
|  | ≥ 65 | 27 | 11 | 477.2 | 0.0231 | 31 | 28 | 738.4 | 0.0379 | 0.45 (0.27-0.77) |  |
| Sex | Male | 39 | 10 | 757.6 | 0.0132 | 44 | 33 | 1185.1 | 0.0278 | 0.40 (0.24-0.67) | 0.854 |
|  | Female | 36 | 14 | 920.7 | 0.0152 | 66 | 46 | 1901.4 | 0.0242 | 0.62 (0.42-0.91) |  |
| *EGFR* mutation type | Exon 21 Leu858Arg | 22 | 10 | 411.9 | 0.0243 | 34 | 29 | 860.8 | 0.0337 | 0.69 (0.42-1.13) | 0.030 |
|  | Exon 19 del | 53 | 14 | 1266.4 | 0.0111 | 76 | 50 | 2225.7 | 0.0225 | 0.46 (0.31-0.69) |  |
| Brain Metastasis | Yes | 39 | 14 | 787.5 | 0.0178 | 30 | 20 | 831.5 | 0.0241 | 0.70 (0.42-1.17) | 0.201 |
|  | No | 36 | 10 | 890.8 | 0.0112 | 80 | 59 | 2255 | 0.0262 | 0.43 (0.29-0.64) |  |

IR, Incident rate; HR, Hazard ratio.

Supplementary Table S4. Subgroup analysis of the risk of progression in patients who received lazertinib or osimertinib after inverse probability of treatment weighting using propensity scores.

| variable | | Lazertinib | | | | Osimertinib | | | | Crude HR | p for interaction |
| --- | --- | --- | --- | --- | --- | --- | --- | --- | --- | --- | --- |
|  |  | n | No of event | Person-year | IR | n | No of event | Person-year | IR |  |  |
| Age group | < 65 | 48 | 36 | 629.3 | 0.057 | 79 | 71 | 1432.5 | 0.050 | 1.17 (0.89-1.54) | 0.694 |
|  | ≥ 65 | 27 | 17 | 336.8 | 0.050 | 31 | 30 | 473.3 | 0.063 | 0.70 (0.46-1.06) |  |
| Sex | Male | 39 | 27 | 459.7 | 0.059 | 44 | 43 | 711.4 | 0.060 | 0.94 (0.66-1.32) | 0.539 |
|  | Female | 36 | 26 | 506.4 | 0.051 | 66 | 58 | 1194.4 | 0.049 | 1.08 (0.80-1.47) |  |
| *EGFR* mutation type | Exon 21 Leu858Arg | 22 | 17 | 240.9 | 0.071 | 34 | 32 | 602.2 | 0.053 | 1.30 (0.85-1.99) | 0.186 |
|  | Exon 19 del | 53 | 36 | 725.3 | 0.050 | 76 | 69 | 1303.7 | 0.053 | 0.93 (0.71-1.23) |  |
| Brain Metastasis | Yes | 39 | 27 | 450.0 | 0.060 | 30 | 27 | 508.9 | 0.053 | 1.10 (0.74-1.64) | 0.511 |
|  | No | 36 | 26 | 516.1 | 0.050 | 80 | 74 | 1396.9 | 0.053 | 0.98 (0.74-1.30) |  |

IR, Incident rate; HR, Hazard ratio.

**Supplementary Table S5**. The number of patients who maintained lazertinib treatment beyond progressive disease and duration of the treatment in the lazertinib group.

|  | Before PSM, Lazertinib 240 mg (n=75) | | After PSM, Lazertinib 240 mg (n=60) | |
| --- | --- | --- | --- | --- |
| **Beyond PD treatment^a^, n(%)** | 19 | (25.3) | 15 | (25) |
| **Duration of treatment beyond PD, n(%)** |  |  |  |  |
| 21 to < 28 days, n | 1 | (5.3) | 0 | (0) |
| 29 to < 90 days, n | 6 | (31.6) | 5 | (33) |
| 91 to < 180 days | 5 | (26.3) | 5 | (33) |
| 181 to < 365 days | 5 | (26.3) | 3 | (20) |
| >= 365 days | 2 | (10.5) | 2 | (13) |
| **Beyond PD treatment duration, days, median (range)** | 158 | (27-474) | 158 | (42-474) |

PD, progressive disease; PSM, propensity score matching.

^a^ Lazertinib was administered over 21 days following disease progression, as assessed by the investigator.
